# Interventional real-time molecular MRI for targeting early myocardial injury in a pig model

**DOI:** 10.1038/s44303-025-00069-z

**Published:** 2025-02-03

**Authors:** Timo Heidt, Simon Reiss, Julien Thielmann, Christian Weber, Alexander Maier, Thomas Lottner, Heidi R. Cristina-Schmitz, Timon Bühler, Diana Chiang, Claus Jülicher, Carolin Wadle, Ingo Hilgendorf, Dennis Wolf, Gavin Tumlinson, Luis Hortells, Dirk Westermann, Michael Bock, Constantin von zur Mühlen

**Affiliations:** 1 Department of Cardiology, Medical Center University of Freiburg, Freiburg, Germany; 2 Faculty of Medicine, University of Freiburg, Freiburg, Germany; 3 Experimental Physics, Department of Radiology, University Medical Center Freiburg, Freiburg, Germany; 4 Center for Models and Transgenic Services - Freiburg (CEMT-FR) - Experimental Surgery, Medical Center University of Freiburg, Freiburg, Germany; 5 Spemann Graduate School of Biology and Medicine (SGBM), University of Freiburg, Freiburg, Germany; 6 Institute for Experimental Cardiovascular Medicine, Medical Center University of Freiburg, Freiburg, Germany; 7 Institute of Experimental and Clinical Pharmacology and Toxicology, Faculty of Medicine, University of Freiburg, Freiburg, Germany

**Keywords:** Magnetic resonance imaging, Molecular imaging

## Abstract

Myocardial ischemia induces tissue injury with subsequent inflammation and recruitment of immune cells. Besides myocardial tissue characterization, magnetic resonance imaging (MRI) allows for functional assessment using molecular imaging contrast agents. Here, we assessed ischemic cardiac lesions non-invasively directly after ischemia/reperfusion (I/R) in a porcine model by advanced MRI techniques and molecular imaging, targeting the cell adhesion molecule P-selectin functionalized with microparticles of iron oxide (MPIO). We used a closed-chest model of I/R by temporary coronary balloon-occlusion, real time 3T MRI-guided coronary injection of MPIO-based contrast agents, as well as injury, edema and iron-sensitive MRI. Within the first hours after I/R, we found T1 mapping to be most sensitive for tissue injury, with no changes in edema-sensitive MRI. Intriguingly, P-selectin MPIO contrast agent selectively enhanced the ischemic area in iron-sensitive MRI. In conclusion, this approach allows for sensitive detection of early myocardial inflammation beyond traditional edema-sensitive imaging.

## Introduction

Myocardial infarction (MI) and heart failure are deadly consequences of atherosclerotic vascular disease^1^. Infarct size defines the risk of functional loss and adverse cardiac remodeling^2,3^. With increasing duration of atherothrombotic coronary occlusion, cardiomyocytes are permanently lost and a transmural cardiac lesion is formed. Early reperfusion therefore plays an essential role in preserving cardiac function, and, thus, constitutes a primary goal in the management of patients with acute MI. Next to time of ischemia, inflammation represents another driving force of wound healing. Dying cardiomyocytes liberate danger-associated molecular patterns that activate endothelial cells and platelets to promote the recruitment of innate immune cells for the removal of necrotic debris, repair or degradation of injured cardiac cells^4^. P-selectin is among the early cell adhesion molecules expressed by activated endothelial cells. In resting condition P-selectin is stored in subcellular vesicles. Upon activation it is rapidly translocated to the cell membrane to promote leukocyte rolling prior to firm adhesion and transmigration. In platelets, P-selectin participates in platelet-leukocyte complex formation that enhance endothelial attachment and migration^5^. After transmigration, monocytes transform to pro-inflammatory macrophages to breakdown debris and injured cells. Based on the local microenvironment immune cells may thereby repair or replace cardiomyocytes. While some degree of inflammation is essential for wound healing, exaggerated presence of immune cells in the lesion may also increase cardiomyocyte removal with detrimental consequences for cardiac function^6^. Assessment of inflammatory activity may therefore enable cardiovascular risk assessment after MI^7^.

Due to the excellent soft tissue contrast, magnetic resonance imaging (MRI) has become the gold standard for non-invasive myocardial tissue characterization. T2-weighted imaging, advanced relaxometric mapping techniques (T1-, T2-, T2* mapping) and contrast-enhanced MRI (late gadolinium enhancement (LGE)) allow for the detection of tissue edema and structural abnormalities as myocardial fibrosis or scarring^8,9^. After MI, LGE depicts the extent and transmurality of the scar^10^. Furthermore, T2-weighted fluid sensitive imaging or advanced mapping helps to delineate the previously ischemic tissue by means of highlighting excess extracellular fluids. While these MRI techniques are well established in clinical routine, they only represent indirect indicators of cardiac inflammation.

Molecular imaging constitutes an intriguing approach to improve diagnostic capabilities of non-invasive imaging tools. Functionalizing gadolinium or iron oxide with target-specific receptor-ligands enables localization of specific contrast deposition thus tissue characterization^11,12^. Previously, molecular MRI has successfully been used in several small animal models targeting diverse receptors of the inflammatory cascade of atherothrombotic disease including cell adhesion molecules^13–15^. In various previous studies, we were also able to image platelet-mediated atherothrombosis and myocardial inflammation in small animal models—and a potential transfer towards large animals would be of interest^16–18^.

With increasing appreciation of the role of inflammation in human cardiovascular disease^19^, methods would be highly desirable that combines the standard assessment of edema and fibrosis with molecular imaging of inflammatory targets in a hybrid imaging approach. So far, the transition of this concept to a larger model or the patient has been restricted to nuclear imaging due to challenges with sensitivity^20^.

In this study we demonstrate that molecular MRI with P-selectin allows to detect early signs of cardiac inflammation after ischemia and reperfusion injury in a pig model.

## Methods

### Animals

All experiments were conducted in accordance with FELASA and GV-SOLAS standards for animal welfare. Experiments were approved by the local ethics committee of Freiburg University and the regional council of Freiburg, Baden-Wuerttemberg, Germany (licence number 35-9185.81/G-21/008).

In total, experiments were performed on 7 juvenile (3 months of age) domestic landrace pigs (body weight 50–70 kg). For premedication, the pigs received an intramuscular injection of midazolam (0.5 mg/kg body weight (bw)) and ketamine (20 mg/kg bw). After preoxygenation, anesthesia was induced by propofol injection (2–4 mg/kg bw) via a peripheral vein catheter and maintained with a mixture of isoflurane (1.5–2%) and oxygen/air (FiO_2_ > 0.3) as well as intravenous (iv) administration of vecuronium (0.2–0.4 mg/kg bw per hour). Fluid loss was compensated at a dose of 5–10 ml/kg bw ringer’s solutionper hour. Analgesia was maintained by intravenous application of fentanyl at a dose of 0.002–0.004 mg/kg bw per hour. Mechanical ventilation (IPPV) was adjusted to keep parameters within physiological range. Oxygen level, electrocardiogram and concentration of carbon dioxide were monitored.

### Closed chest model of myocardial ischemia/reperfusion injury

For angiography, a 10 French arterial access sheath was introduced into the right femoral artery using ultrasound needle-guidance. To reduce the risk of sudden fatal arrhythmias, potassium and magnesium were supplemented and amiodarone (10 mg/kg bw) was intravenously administered prior to the procedure. Coronary angiography was performed using a C-arm x-ray (Philips Medical) and standard coronary catheters. The left coronary artery was depicted with bolus injection of diluted iodinated contrast agent (Accupaque^TM^ 300 mg, GE Healthcare). A coronary wire was thus mounted with a balloon catheter and inserted into the circumflex coronary artery. The balloon was then inflated in the mid-segment of the coronary artery to trigger ischemia, and contrast agent was injected to ensure tight sealing of the vessel. After 40 min, the balloon was deflated and removed from the coronary vessel. Then, the study animal was transferred to the MRI.

### Molecular contrast agent

Construction of the molecular contrast agent was performed using pelleted MyOne^TM^ Tosylactivated MPIOs with a size of 1 µm. MPIO pellets were washed in 0.1 M natrium borate buffer and resuspended in ammonium sulfate buffer containing 200 micrograms of antibody (either mouse-anti-pig P-selectin antibody or mouse-anti-pig IgG antibody) to reach a final concentration of 1 M for 20 h at 37 °C. Constant rotation ensured the separation of pelleted beats. Afterwards, residual active tosyl-remnants were removed using a blocking buffer and contrast agent was resuspended in a storage buffer with constant rotation.

### In vitro flow chamber

Cell culture dishes (35 mm; CytoOne) were coated with 1 ml fibrinogen (100 µg/ml) and stored overnight at 4 °C. The following day human blood was collected in a citrate tube and separated in various blood components via centrifugation at 150 G for 5 min. The supernatant (platelet-rich plasma; PRP) was then transfered into a Falcon tube and 1 ml of PRP was applied to each of the previously prepared fibrinogen-coated dishes to allow platelet adhesion to the surface of the dish. Platelet activation was induced using a 1:10 dilution of 20 µg of adenosine diphosphate (ADP). The activation process was carried out for a precisely controlled duration of 30 min at room temperature.

The parallel plate flow chamber kit (GlycoTech, Rockville, Maryland, USA) consists of a transparent chamber with two parallel plates, one plate is coated with a monolayer of platelets, and the other plate is a continous fluid flow channel. A syringe pump connected to the inlet port is used to deliver a precise and constant flow of contrast agent through the chamber and thus over the platelets. The outlet port is used to allow the left over contrast agent to exit the chamber into a waste container. The chamber was observed using a microscope (Zeiss Vert. A1) at ×20 magnification connected to a digital imaging system (AxioCam ICc1, Carl Zeiss AG, Feldbach, CH).

IgG-MPIO served as a non-specific control group, whereasanti-CD62P-MPIO was used as a contrast agent with specific binding properties for P-selectin. The binding properties of both contrast agents were evaluated on the platelet monolayer over a specific duration of 60 s. The video recording commenced upon the appearance of the initial MPIO within the designated high power field, measuring 450 µm × 350 µm. Only MPIOs that demonstrated adhesion for a minimum of 10 seconds were deemed to have established a bond with the platelets.

### Incubation assay

Porcine endothelial cells were cultured according to the manufacturer’s protocol (Sigma Aldrich Chemie GmbH, P300-05). In short after thawing the cryovials in a 37 °C water bath, the cells were resuspended a 10% dulbecco’s modified eagle medium (DMEM). The cells were centrifuged, washed three times with DMEM, and finally resuspended in Porcine Endothelial Growth Medium. The cell suspension was transferred to a T-75 cell culture flask, and daily medium changes were performed until the cells reached 60% confluency. The volume of the culture medium was then doubled, and after further incubation, the cells were split onto 12-well-dishes (Thermo Fisher Scientific, 168844). For proper comparison of the specifically targeting P-Selectin-MPIO contrast agent we compared the binding properties to MPIO with unspecific targeting. For each contrast agent six dishes were seeded with a total of 4000 cells each. Each contrast agent was prepared identically as described above. 10 µl of each contrast agent was diluted with 990 ml of PBS and incubated for 30 seconds. After each dish was washed thoroughly with PBS. For subsequent analysis photographic documentation was performed to capture the different binding properties of each contrast agent. Per dish 10 photos of evenly distributed cells were taken randomly yielding a total of 60 photos per group. Each MPIO located exactly next to or on top of a cell was considered as bound to the endothelial cell layer.

### Immunofluorescence staining

For each heart sample, three specimens of each supply areas of the RCA, LCX, and LAD (nine in total) were collected from porcine myocardium and cryo-embedded for further histologic processing. Using a microtome, cryosections of 6 µm were extracted and stained with hematoxylin (25%). 10 μm sections were cut for immunofluorescence imaging (for quantification, at least 9 sections per heart were used). Slices were permeabilized with 0.1%Triton X-100 (Invitrogen, Waltham, MA, USA) at RT for 15 min. Antigen retrieval was performed by boiling in 1× citrate buffer-based antigen retrieval solution (H-3300, Vector laboratories, USA). Unspecific antibody binding was blocked with 6% donkey serum (Sigma-Aldrich, Waltham, MA, USA), and 2.5% bovine serum albumin (BSA, Sigma-Aldrich, Waltham, MA, USA) at RT for 1 h. Samples were incubated with a primary mouse antibody anti-P-selectin (NB100-65392, Novus Biologicals, C), USA) at 4 ^o^C overnight. After washing, samples were incubated with Alexa Fluor 555 conjugated donkey anti-mouse secondary antibody at 1:400 for 1-h at RT. DAPI was applied at 1:500 concentration in PBS for 10 min. Samples were mounted with Fluoromount-G (ThermoFisher, Waltham, MA, USA). Samples were imaged with an automated slide scanner (AxioScan.Z1 Zeiss, Jena, Germany).

For image analysis, thresholds were manually selected in Zen Blue microscopy software (Zeiss, Jena, Germany). To acquire a baseline for fluorescence, a value eliminating approximately 95% of nonzero p-selectin pixels remote sample images was chosen and applied. Autofluorescence from the green channel was used to determine the total area of the tissue. A custom Python script using the packages czifile (Christoph Gohlke, University of California, Irvine) and NumPy was used to quantify fluorescent area. Positive pixels for autofluorescence were compared to positive pixels for p-selectin (red channel) in a given sample. Coverage was determined by dividing the sum of pixels both positive for P-selectin and autofluorescence by the sum of all total membrane pixels positive for autofluorescence.

### CMR

Cardiac MR imaging and MR-guided coronary catheterization were performed at a clinical 3T system (PrismaFit, Siemens) with the animals in head-first supine position and the heart at magnet iso-center. A 32-channel spine coil and an anterior 18-channel thorax coil array were used for signal reception. An ECG with four leads was attached and to enable cardiac gating of the imaging sequences. All acquisitions except the cine and real-time sequences were gated to end-diastole. Real-time images for catheter guidance were displayed on an in-room monitor (BOLD Screen 24, Cambridge Research Systems Ltd) positioned close to the patient table and communication between the cardiologist and the system operator was established via the conventional headphones and in-room microphone of the MRI system. A custom-made active coronary guiding catheter equipped with a single-loop receive coil at the tip was used for catheterization of the left coronary artery. The catheter was connected to the MRI system via a custom-made tuning/matching circuit with variable signal attenuation. First functional images were recorded approximately 2 h after reperfusion. A diagram of the experiments with a typical timeline of the procedures and image acquisitions is shown in supplement Fig. 1.

### Functional imaging

Cardiac MRI started with the acquisition of a set of localizer images in the three orthogonal standard views and definition of the main axes of the hearts. Then, a multi-slice 2D cine bSSFP sequence was acquired in short-axis view for functional and volumetric imaging (TE/TR = 1.5/3.0 ms, flip angle (FA) = 42°, BW = 970 Hz/px, FoV = 340 × 273 mm², matrix: 224 × 126, slice thickness (SL) = 8 mm, number of slices: 6–8, retrospective cardiac gating with 20 reconstructed phases, multiple breath-holds).

### T1, T2 and T2* mapping

Parametric mapping of the relaxation times T1, T2, and T2* was performed in 3 or 4 mid-ventricular short axis slices depending on the size of the heart about 2 to 2.5 h after reperfusion. T1 maps were acquired with an inversion recovery bSSFP sequence (TE/TR = 1.2/2.7 ms, FA = 35°, BW = 1085 Hz/px, FoV = 360×307 mm², matrix: 192 × 132, SL = 5 mm, 8 different TI values from 100 to 4500 ms depending on the heart rate, single breath-hold). T2 maps were acquired with a T2-prepared FLASH sequence with varying T2 preparation times (TE/TR = 1.3/3.1 ms, TE_T2prep_: [0, 30, 40] ms, FA = 12°, BW = 1185 Hz/px, FoV = 360 × 247 mm², matrix: 192×132, SL = 6 mm, single breath-hold). The inline motion correction and calculation of T1/T2 values provided by the vendor were used for both T1 and T2 mapping. T2* mapping was performed with a multi-echo FLASH sequence (TE/TR = [2.4, 6.0, 9.5, 13.0]/16.2 ms, FA = 12°, BW = 590 Hz/px, FoV = 260² mm², matrix: 256², SL = 5 mm, single breath-hold) and T2* maps were calculated offline in Matlab by a pixel-wise linear fit of the logarithm of the signal intensities. The T2* mapping sequences were acquired again after the injection of MPIOs under real-time guidance.

### Real-time MRI catheter guidance

After functional and parametric imaging a non-contrast 3D compressed-sensing accelerated prototype whole heart FLASH sequence (TE/TR = 2.3/5.2 ms, FA = 15°, BW = 250 Hz/px, FoV = 320x310x139 mm³, matrix: 256 x 248 x 104, navigator gating to end-expiration) was acquired for coronary angiography and planning of the imaging planes for the MR-guided catheterization. Therefore, three planar views were extracted from the 3D dataset to cover the aortic arch and the left coronary ostium in a short-axis and long-axis view. Catheterization of the LCA was performed under imaging with a real-time FLASH sequence (TE/TR = 1.3/3.4 ms, FA = 10°, BW = 790 Hz/px, FoV = 289² mm², matrix: 192×144, SL = 8 mm) 4 h after reperfusion. Using an in-room video monitor (BOLD Screen 24, Cambridge Research Systems Ltd, Rochester, UK) next to the patient table of the MRI system, the real-time images were presented to the interventionalist during MRI. Conventional headphones and the in-room microphone of the MRI system were used for communication between the interventionalist and the system operator. First, a guidewire (standard Terumo 0.018”) was inserted via the arterial sheath to guide a custom-made active 6 F guiding catheter (Terumo; Optitorque Radial Tig II 4.0) to the aortic root. The guiding catheter equipped with a single-loop receive coil at the tip was used for catheterization of the left coronary artery. The catheter was connected to the MRI system via a custom-made tuning/matching circuit with variable signal attenuation. Successful intubation was verified by imaging the perfusion of a small amount of 1% Gd-solution (1:20, Gd-DTPA, Magnevist, Bayer, Germany) injected via the catheter in a single short-axis slice with an inversion recovery FLASH sequence (TE/TR = 1.0/2.0 ms, TI = 95 ms, FA = 10°, BW = 1185 Hz/px, FoV = 360 × 270 mm², matrix: 192 × 106, SL = 8 mm, single breath-hold). Molecular contrast agents were injected via the guiding catheter in a volume of 20 ml and flushed with saline. The injection was imaged with a FLASH sequence in short-axis view (TE/TR = 4.0/5.0 ms, FA = 12°, BW = 1185 Hz/px, FoV = 280² mm², matrix: 128×102, SL = 6 mm, single breath-hold).

### Real-time imaging

After functional and parametric imaging a non-contrast 3D compressed-sensing accelerated prototype whole heart FLASH sequence (TE/TR = 2.3/5.2 ms, FA = 15°, BW = 250 Hz/px, FoV = 320x310x139 mm³, matrix: 256 × 248 × 104, navigator gating to end-expiration) was acquired for coronary angiography and planning of the imaging planes for the MR-guided catheterization. Therefore, three planar views were extracted from the 3D dataset to cover the aortic arch and the left coronary ostium in a short-axis and long-axis view. Catheterization of the LCA was performed under imaging with a real-time FLASH sequence (TE/TR = 1.3/3.4 ms, FA = 10°, BW = 790 Hz/px, FoV = 289² mm², matrix: 192×144, SL = 8 mm) 4 h after reperfusion. Successful intubation was verified by imaging the perfusion of a small amount of 1% Gd-solution injected via the catheter in a single short-axis slice with an inversion recovery FLASH sequence (TE/TR = 1.0/2.0 ms, TI = 95 ms, FA = 10°, BW = 1185 Hz/px, FoV = 360 × 270 mm², matrix: 192 × 106, SL = 8 mm, single breath-hold). The MPIO contrast agent was injected after successful intubation and the injection was imaged with a FLASH sequence in short-axis view (TE/TR = 4.0/5.0 ms, FA = 12°, BW = 1185 Hz/px, FoV = 280² mm², matrix: 128 × 102, SL = 6 mm, single breath-hold).

### Real-time MRI catheter guidance

Using an in-room video monitor (BOLD Screen 24, Cambridge Research Systems Ltd, Rochester, UK) next to the patient table of the MRI system, the real-time images were presented to the interventionalist during MRI. Conventional headphones and the in-room microphone of the MRI system were used for communication between the interventionalist and the system operator. The animal was positioned on the MR table in a supine position with the heart located in the magnet’s isocenter. For a full coverage of the vascular system, a posterior 32-channel spine coil and an anterior 18-channel thorax coil array were used. The wireless ECG system supplied by the vendor was attached with hydrogel electrodes.

After the acquisition of an initial set of localizer images, a 3D whole-heart ECG-triggered gradient echo (FLASH) data set was acquired with the following imaging parameters: fat saturation, TE/TR = 1.6/3.5 ms, FA = 16°, FoV = 282x282x102 mm³, matrix: 176x176x64, T2-preparation with TE_T2prep_ = 40 ms, GRAPPA acceleration factor *R* = 2. The ECG-triggered FLASH sequence was repeated later during the experiment to confirm the position of the interventional instruments.

First, a guidewire (standard Terumo 0.018”) was inserted via the arterial sheath to guide a modified 6 F guiding catheter (Terumo; Optitorque Radial Tig II 4.0) to the aortic root. After intubation of the LCA, proper position of the guiding catheter was confirmed by injection of 5 ml diluted gadolinium contrast agent (1:20, Gd-DTPA, Magnevist, Bayer, Germany) via the guiding catheter. A 2D real-time radial bSSFP sequence with the following imaging parameters was used to monitor the advancement of the instruments: TE/TR = 1.4/2.8 ms, #spokes = 105, FA = 40°, FoV = 275 × 275 × 7 mm³, matrix: 160 × 160, fat saturation. Real-time image slice orientations and positions were defined using the localizer and 3D FLASH images acquired prior to the catheter advancement. Molecular contrast agents were injected via the guiding catheter in a volume of 20 ml and flushed with saline. Out of the 7 animals we investigated, 3 pigs were treated with P-selectin-MPIO and 3 pigs with control-MPIO, respectively. One animal died of ventricular fibrillation shortly before intracoronary injection of the contrast agent.

### LGE imaging

Late gadolinium enhancement (LGE) image data were acquired after the coronary catheterization and 10 min after intravenous injection of 2.5 mmol/kg Gd approximately 4 h after reperfusion. A TI scout was first acquired in short axis view to determine the optimal inversion time. The LGE sequence was then acquired with phase sensitive inversion recovery FLASH sequence in short-axis view (TE/TR = 1.4/3.7 ms, FA = 20°, BW = 465 Hz/px, FoV = 360² mm², matrix: 144×141, SL = 10 mm, single breath-hold).

### Post-processing

Dedicated MRI post-processing software (CVI 42, Circle Imaging) was used to quantify T2* times from 3 short axis slices of the basal, mid and apical left ventricle. Regions of interest (ROI) were defined in the area of LAD, LCX or RCA on each slice. For in vivo analysis of the mean R2* values in each ROI was calculated and the difference between the values measure after and before MPIO injection are used as a measure for the amount of MPIO in each region.

For ex vivo R2* maps, the number of pixels with high R2* values was counted. Therefore, a normal distribution was fitted to the histogram of R2* values of the whole LV myocardium. Then, all pixels with an R2* value greater than the mean of the fitted distribution plus two times the standard deviation were counted for both the LCX and LAD area. These numbers were normalized to the area of the LCX and LAD, respectively and the ratio of these normalized values was calculated.

### Ex vivo R2* mapping

Ex vivo imaging was performed after the hearts were removed and fixed in formaldehyde (4% PFA) for at least 7 days to allow for the fixative to fully diffuse through the heart. The hearts were imaged at the same 3 T MRI system as the in vivo experiments. For imaging, the hearts were placed in a plastic container containing the fixative positioned at iso-center inside a 64 channel head/neck coil. Three-dimensional R2* maps were acquired via a multi-echo spoiled gradient echo sequence with 0.58 mm isotropic resolution covering the whole heart (TE/TR = [3.4, 9.8, 17.1]/23 ms, FA = 12°, BW = 260 Hz/px, FoV = 129 × 129 × 84 mm³, matrix: 224 × 224 × 144, averages = 2). R2* maps were calculated offline in the same way as the in vivo R2* maps.

### FACS

For flow cytometry full blood was stained with antibodies targeting cell specific epitopes after red blood cell lysis. Neutrophils were identified according to SSC and FSC, classical-type monocytes were identified as SWC3^+^, CD163^neg^, CD14^+^, non-classical-type monocytes were identified as SWC3^+^, CD163^+^, CD14^int^. Analysis was performed using FACS Diva software.

#### Hematoxylin staining

For analysis of MPIO, hematoxylin staining was performed using a 25% hematoxylin solution for 50 s. Further washing steps with saline were held to a minimum to avoid loss of MPIO binding. For analysis a bright-field microscopy at ×100 magnification was used. 20 high power fields (HPF) were recorded in a standardized fashion and analyzed to count individual MPIO. In total, 3 slides per region of interest (RCA, LCX, and LAD) were analyzed covering the epicardium, mid-section and subendocardium.

### Statistics

GraphPad Prism software (GraphPad Software, Inc.) was used for statistical analyses. Results are depicted as mean ± standard error of mean. For two-group comparison a Mann–Whitney *U* test for nonparametric data or Students’s *t* test for parametric data was used. For a comparison of more than two groups one-way ANOVA, followed by a Bonferroni test for multiple comparison, was applied. *P* values of *p* < 0.05 indicate statistical significance.

## Results

### 40 min of I/R induces early injury only visible by myocardial T1 mapping

**Fig. 1:**
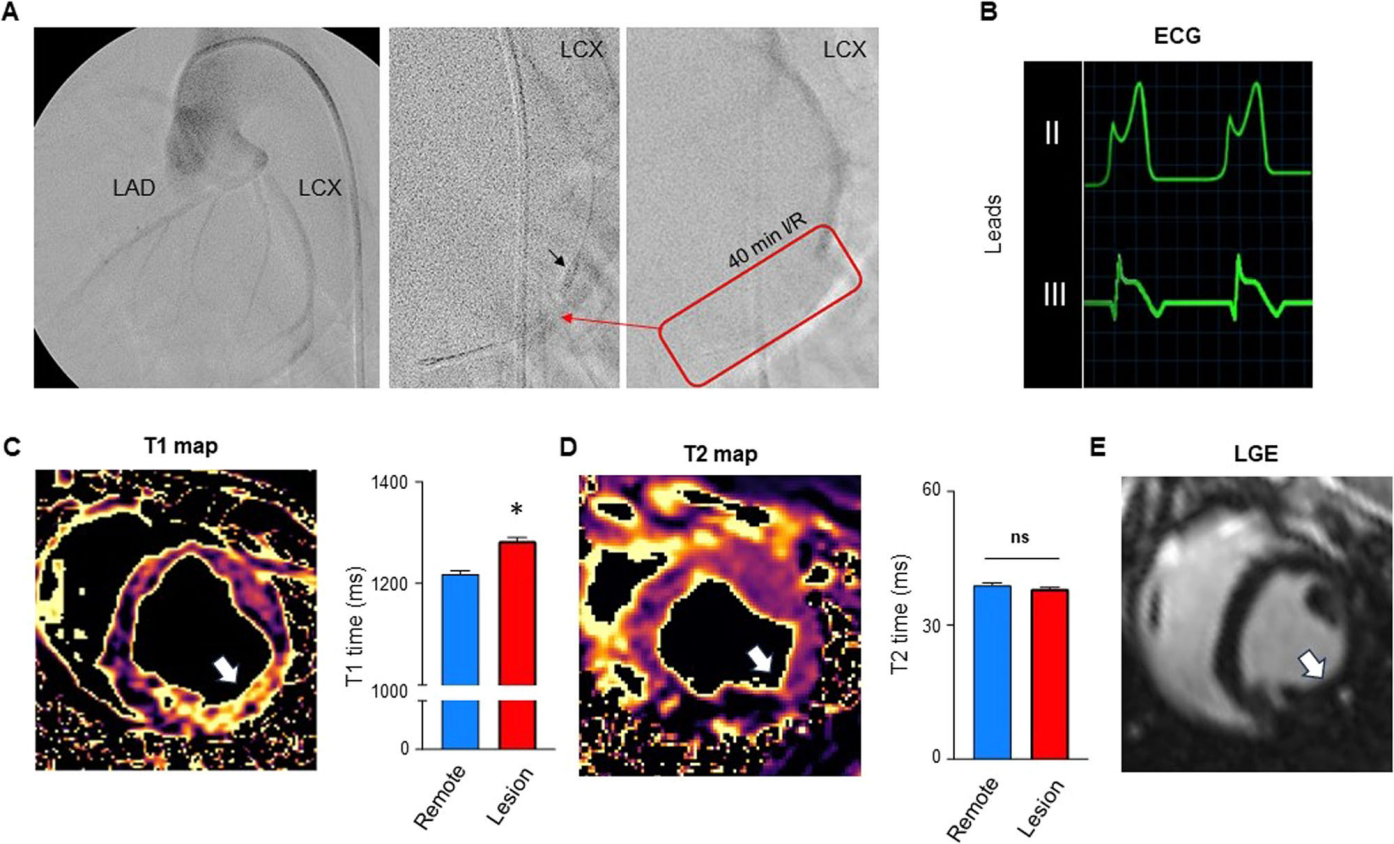
Ischemia and reperfusion injury. **A** Coronary angiography of the left coronary artery. **Left** Overview of the left anterior descending (LAD) and left circumflex artery (LCX). **Middle** A standard coronary wire (red arrow) and obstructive coronary balloon (black arrow) are introduced into the LCX. Then the balloon is inflated to block blood the supply to the distal myocardium. **Right** Red box indicates the blocked LCX artery during contrast deposition. After 40 min of ischemia the balloon is removed for reperfusion. **B** ECG showing inferior leads II and III with ST-segment elevations during ischemia. **C** T1 mapping 2–4 h after reperfusion indicates a significantly elevated T1 time in the “Lesion” area of the LCX as compared to “Remote” (LAD/RCA area); *n* = 72 values per group, *p* < 0.001. Note, that in each of the 7 animals 3–4 short axis views were acquired with multiple repetitions resulting in 72 measurements. The arrow indicated the area of the lesion. **D** T2 mapping 2–4 h after reperfusion shows no difference between “Lesion” and “Remote”; *n* = 72 values per group, *p* = 0.11. The arrow indicated the area of the lesion. **E** After 40 min of ischemia and 4 h of reperfusion, no LGE can be detected in the “Lesion” area; *n* = 6. The arrow indicated the area of the lesion. *p < 0.05, Mann–Whitney *U* test. **F** The left ventricular ejection fraction was reduced in both groups (control MPIO 43.0 ± 4.6%, P-Selectin MPIO 47.6 ± 5.1%) without a significant difference (*p* = 0.30; Mann–Whitney *U* test)

To investigate early changes after myocardial injury we used a closed-chest model of I/R placing an obstructing coronary balloon into the circumflex (LCX) coronary artery (Fig. 1A) Ischemia was confirmed by documentation of significant ST-segment elevation on the corresponding monitor ECG (Fig. 1B). In addition, echocardiography confirmed ischemia by wall motion abnormalities (Supplement Movie 1, 2). Reperfusion was obtained after 40 min of ischemia and animals were directly transferred to the MRI. Due to the time of ischemia and transportation first images were acquired 2 h after onset of ischemia. Performing advanced MR imaging, T1 mapping indicated elevated T1 times within the lesion compared to remote areas (*p* < 0.001, Fig. 1C). However, edema sensitive T2 mapping remained unchanged (*p* = 0.11, Fig. 1D). Likewise, late gadolinium enhancement was absent 4 h after onset of ischemia in all animals (Fig. 1E). The left ventricular ejection fraction was similarly reduced in both groups (Fig. 1F).

### Inflammation responds early after myocardial ischemia

**Fig. 2:**
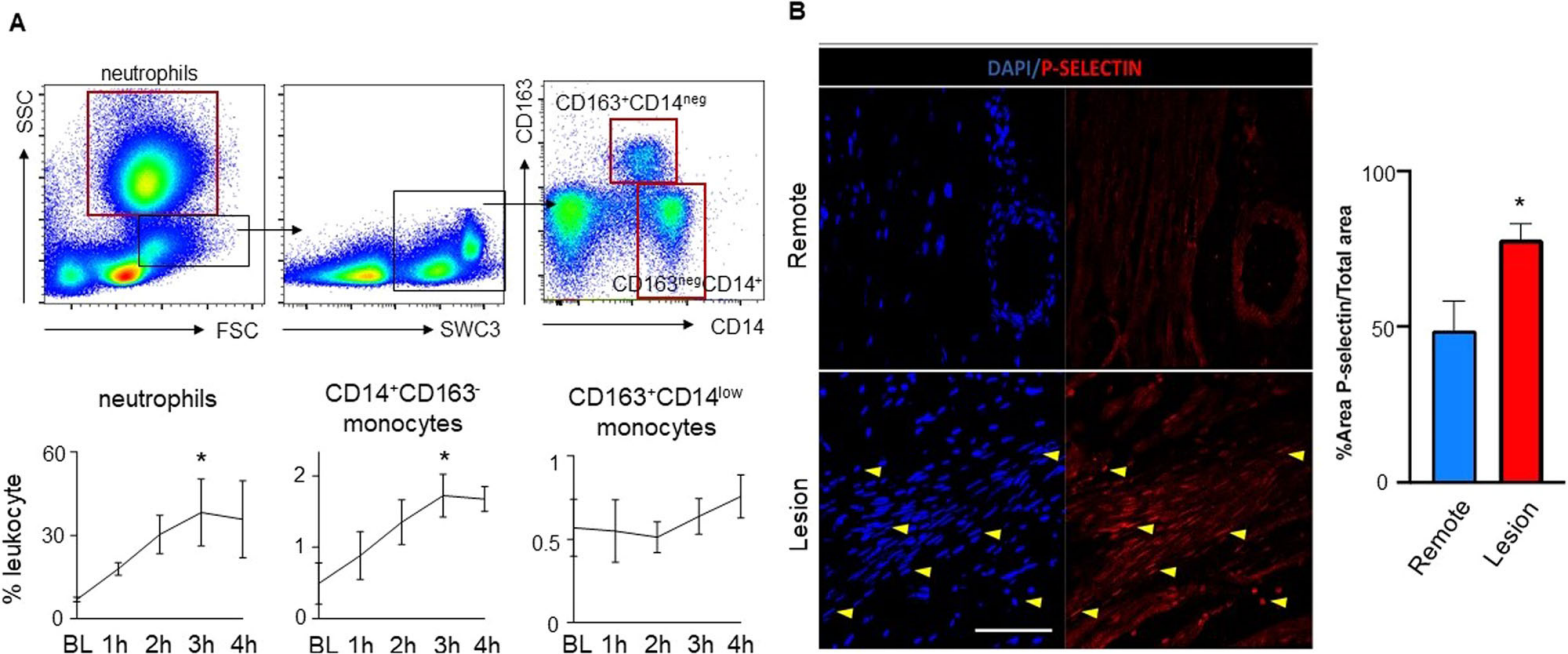
Post-ischemic inflammation. **A** Flow cytometry characterization of innate immune cells in the blood after I/R. **Above** FACS plots indicate the gating scheme for neutrophils and monocyte subsets. Red boxes indicate final cell populations, while black boxes were used for further subtype characterization. **Below** Timeline analysis of innate immune cells from baseline to 4 h after onset of ischemia for neutrophils, classical-typed CD14+CD163neg monocytes and nonclassical-typed CD14negC163+ monocytes. *p < 0.05 for the comparison of the time point at 3 h versus baseline; *n* = 3 per group, Welch’s unpaired *t* test. **B** Immunofluorescence staining of cardiac tissue from “Lesion” and “Remote” targeting P-selectin expression (red) and nuclei (dapi-blue). After injury, P-selectin expression is significantly increased in the “Lesion” area compared to “Remote”; *n* = 6 per group, *p* = 0.02. Yellow arrows highlight examples of positive P-selectin or DAPI staining. *p < *0.05;* Mann-Whitney *U* test.

To characterize the early inflammatory response after I/R, we analyzed repetitive blood samples at baseline and after ischemia. Flow cytometry analysis of innate immune cells indicated an immediate and steady increase of pro-inflammatory neutrophilic granulocytes and inflammatory “classical-type” CD14^+^ monocyte within the first 3 h after ischemia before reaching a plateau at 4 h. Other “non-classical type” monocyte subsets (CD14^neg^, CD163^+^) remained unchanged (Fig. 2A). In accordance with increased myocardial T1 mapping times and a proinflammatory state in circulation, we detected elevated expression of the leukocyte binding integrin P-selectin within the myocardial lesion area when compared to remote myocardial tissue (*p* = 0.02, Fig. 2B).

### P-selectin expression by platelets and endothelial cells can be selectively targeted with a functionalized molecular imaging contrast agent

**Fig. 3:**
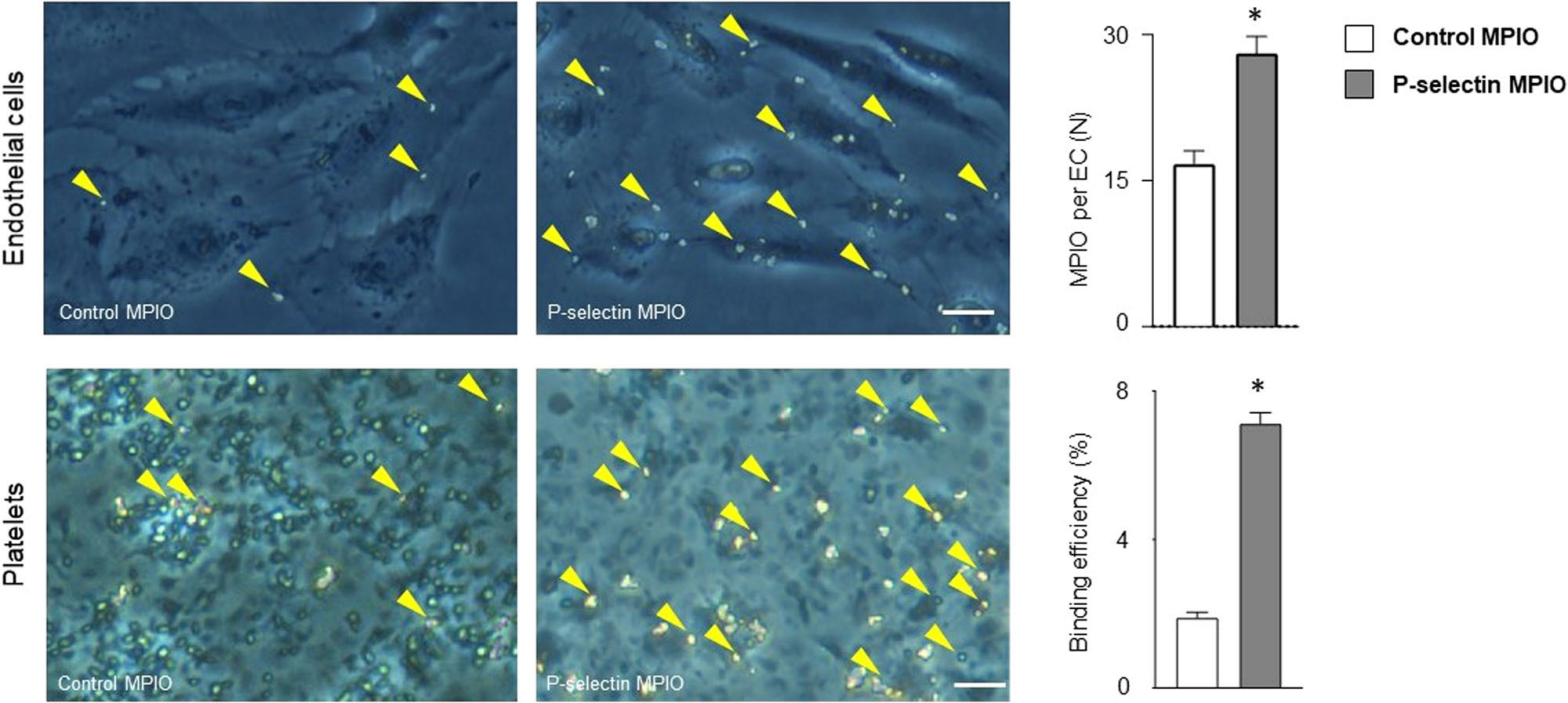
Targeting P-selectin in vitro. Binding of functionalized MPIO P-selectin (Psel-MPIO) to platelets and endothelial cells was evaluated using an incubation assay for endothelial cells (*n* = 60 per group, above) or an in vitro flow chamber model for platelets (*n* = 6 for P-selectin MPIO versus *n* = 3 control MPIO, below) in vitro. For comparison we used functionalized MPIO targeting unspecific IgG (control MPIO). Both essays confirmed enhanced binding of P-selectin MPIO to platelets (*p* < 0.0001) or endothelial cells (*p* < 0.0001) in comparison to control MPIO. Yellow arrows indicate MPIO. *p < 0.05; Mann–Whitney *U* test.

After MPIO-labeling of P-selectin antibody (P-selectin MPIO) or unspecific IgG antibody (control MPIO), we tested binding efficiency of the contrast agents using an in vitro flow chamber model for platelets or an incubation assay for endothelial cells. In comparison to labeled IgG control, P-selectin contrast agent selectively bound to endothelial cells in vitro and indicated a higher binding efficiency to platelets in a flow chamber model (*p* < 0.0001 for each, Fig. 3).

### In vivo molecular imaging of P-selectin in myocardial I/R

**Fig. 4:**
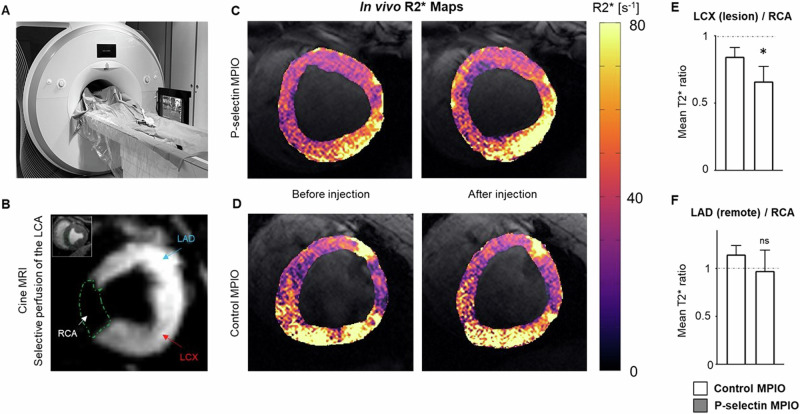
Targeting P-selectin in the ischemic lesion in vivo. **A** Interventional MRI setup for selective contrast injection into the left coronary artery. **B** Perfusion map of the porcine heart. Cine image (corner) shows a midventricular short axis slice of the heart. Gadolinium injection into the left coronary artery (LCA) depicts the supply area of the left circumflex (LCX) and left anterior descending (LAD) arteries as well as the non-perfused area of the right coronary artery (RCA). **C**, **D** T2* maps of the left ventricular short axis after injection of P-selectin MPIO or control MPIO (above: P-selectin MPIO; below: control MPIO). **E**, **F** Injection of P-selectin MPIO induced a significant increase of R2* in the LCX (lesion) compared to RCA (*p* = 0.03), whereas no difference was observed when comparing the equally perfused but non-injured LAD area with RCA area. The control MPIO did not lead to a significant increase in R2* in the LCX and LAD compared to the RCA areas.

Molecular imaging in large animal models is especially challenging due to the large blood pool of the animal. We therefore used interventional MRI to selectively inject MPIO contrast agent into the left coronary artery. Figure 4A depicts the interventional MRI setup. After intubation of the LCA, selective injection of diluted gadolinium allowed to separate the area of the perfused left and non-perfused right coronary artery (Fig. 4B). After injection of P-selectin MPIO, we detected a visible signal decrease in the T2* map indicating shorter T2* times in the lesion area (Fig. 4C, D). Comparing the R2* differences, P-selectin MPIO lead to a significant increase in R2* in the lesion compared to the RCA area (*p* = 0.03, Fig. 4E). This difference, however, was absent when comparing the equally perfused but non-injured LAD area to the RCA. The control-MPIO did not lead to a significant R2* increase for both the lesion and the LAD area.

### Ex vivo MRI and histology confirms selective contrast deposition

**Fig. 5:**
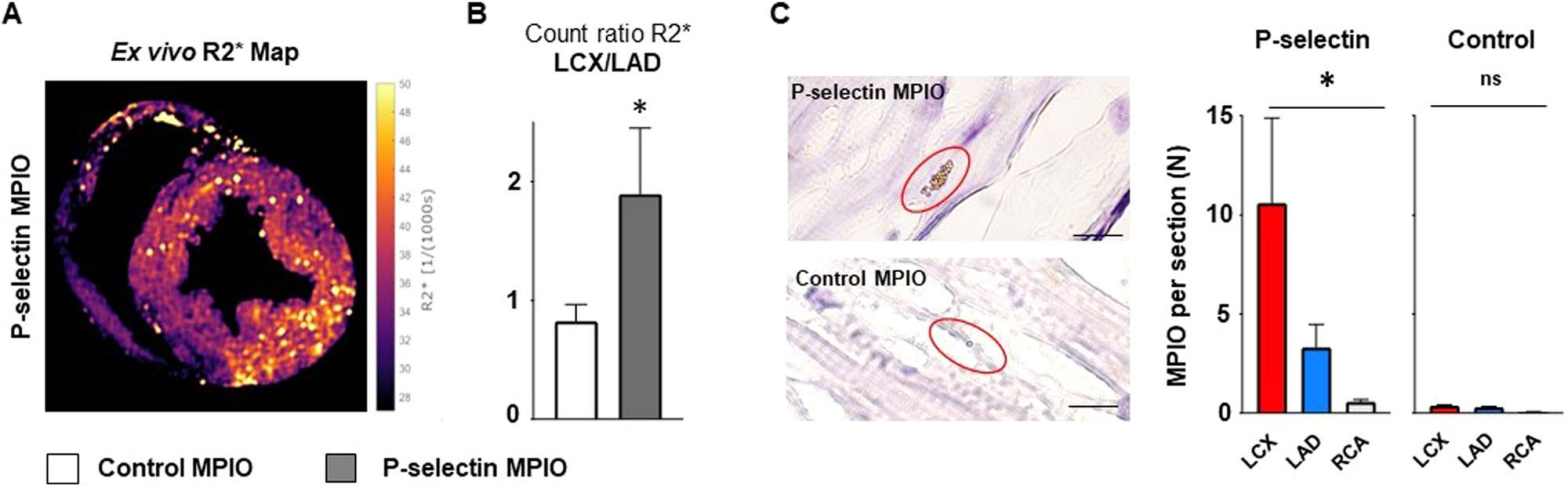
Ex vivo analysis of heart specimens. **A** Ex vivo, high resolution inverse T2* (R2*) map of the left ventricle after I/R of the LCX and injection of P-selectin MPIO perfusion via the left coronary artery. **B** P-selection MPIO selectively enriched in the area of the LCX as compared to control MPIO, *n* = 3 each, *p* = 0.03; standard *t* test, as seen in an increased count ratio which compares the R2* values in ischemic and normal myocardial tissue (see “Post-processing”). **C** We randomly assessed tissue sections taken from specimens resected from the LCX, LAD or RCA area. P-selectin MPIO was found most frequent in the area of the lesion compared to perfused, non-injured LAD area or non-perfused, non-injured RCA area, *n* = 540 HPF (high power fields) per totel area. Only minimal MPIO moieties were found in the tissue after injection of control-MPIO with unspecific binding, *n* = 518 HPF (high power fields) total per area, *p < 0.05 one-way ANOVA.

After termination of the experiment we excised the porcine heart and repeated T2* mapping of the fixed heart ex vivo. Signal inversion (R2*) helped to identify contrast agent deposition in myocardial tissue. In support of our in vivo findings, we detected a significant increase in the signal intensity within the lesion area in comparison to the perfused but non-injured LAD area and the non-perfused RCA area (*p* = 0.03, Fig. 5A). Similar findings were observed when counting MPIO particles in histology sections. In animals injected with P-selectin MPIO we detect highest amounts of MPIO in the lesion area in comparison to the perfused but non-injured LAD area and the non-perfused RCA area, while MPIO target binding was equally low in all areas after injection of control-MPIO (Fig. 5B).

## Discussion

In this study, we demonstrate that molecular targeting of early markers of inflammation can complement existing MRI tools for tissue characterization after ischemic injury providing target-specific information. Importantly, in our study, the application of molecular contrast agents was performed using a real time MRI-guided intervention by selectively injecting the contrast agent into the coronary artery.

Ischemic cardiac injury usually forms a continuum with increasing severity over time^21^. As cardiomyocytes fully depend on oxygen to cover their excessive energy demand, even short durations of MI may induce cell death and apoptosis^22^. With increasing time of impaired myocardial blood flow, injury may expand and reach a critical mass of cardiomyocyte loss for permanent impairment of cardiac function and formation of a non-contractile scar. However, this detrimental development can be interrupted at any time by reperfusion to salvage injured cardiac cells^23^. Next to the necrotic core zone, this consequently leaves an area of unknown fate with cells that are unequivocally lost intermingling with cells that could potentially be saved and repaired, termed ‘area at risk’^24^. Innate immune cells recruited to the lesion have been recognized to be essential in repair or replacement after injury and excessive inflammation has been linked to increased cardiomyocyte loss^25,26^. Likewise, several preclinical studies have demonstrated a beneficial role of immunomodulation to improve wound healing and prevent from adverse cardiac remodeling^27,28^. Highly sensitive and quantitative assessment as well as characterization of inflammation could thus set grounds for individualized risk prediction and novel therapeutic strategies after ischemic injury.

MRI evolved as the gold standard for cardiac tissue characterization due to its capabilities of tissue specific measurement of T1, T2, and T2* relaxation times as well as assessment of gadolinium contrast distribution^29–31^. These techniques have proven to be of value in assisting the non-invasive detection of inflammatory cardiac disease like myocarditis. In myocarditis, T1 mapping demonstrated highest sensitivity for cardiac injury while edema-sensitive T2 mapping can be used to differentiate acute from healing myocarditis^32^. After MI, T1/T2 mapping or late gadolinium enhancement (LGE) are used for quantification of the ischemic scar and the area at risk^33^. In our model we allowed for reperfusion after 40 min of MI. Time of ischemia has been restricted to 40 min to ensure hemodynamic stability. Interestingly, after this time we neither found signs of LGE nor edema-specific increase in T2 times in the lesion up to 4 h after reperfusion which is an indicator of the absence of myocardial scarring or relevant edema. Only T1 mapping detected reproducible alterations in T1 times within the lesion supporting existing evidence of the high sensitivity of T1 mapping in cardiac injury. Yet, reasons for T1 alterations are manifold including fibrosis and infiltrative diseases as well as myocardial edema. Therefore, more specific information is needed.

Following the increase of innate immune cells in the blood early after ischemia, we investigated if activation of inflammatory pathways could be associated to changes in T1. Intriguingly, we found higher expression of the integrin P-selectin in areas with elevated T1 time. P-selectin is mainly expressed by endothelial cells and platelets and is involved in the recruitment process of immune cells to the site of injury^34,35^. In endothelial cells P-selectin induces rolling of leukocytes at the vascular surface prior to firm adhesion and transmigration. In platelets, P-selectin contributes to complex formation with leukocytes to boost immune cell recruitment^36^. Non-invasive detection of P-selectin in cardiac lesions or other inflammatory disease could thus serve as an early and highly specific marker of cardiac inflammation. Yet, little is known about the dynamics of P-selectin expression over time or its relevance for cardiac outcome. In its soluble form, P-selectin was shown to be a predictor of adverse atherosclerotic plaque progression^37^. Whether this is transferable to myocardial injury remains elusive.

Using a covalent binding strategy, we functionalized MPIO with antibodies targeting P-selectin or unspecific IgG as a control compound. The concept of targeted contrast agents for molecular imaging emerged from nuclear imaging techniques and was later transferred to MRI in several preclinical settings. In previous biodistribution experiments, we found MPIOs in lungs, kidneys, liver and spleen 30 min and 24 h after intravenous injection in mice, suggesting rapid clearance^38^. Clearance from vascular receptors already starts much earlier, with peak binding timepoints 45 min after injection^16–18,39^. In all of our previous studies, there were no signs of acute or intermediate toxicity in the tested species (mice, pigs).

Iron oxide compounds are suitable contrast agents due to the strong field distortions that surpass the actual particle size and thus allow for visualization of even smallest moieties. T2* mapping or its inverted R2* map are sensitive to display iron-induced field distortions. In general, MR relaxometry has been applied for various cardiac applications such as the detection of changes in the microvascular structure in hypo-perfused areas by T2* measurements^40,41^, or the quantification of the degree of amyloidosis in the myocardium in T1 and T2 parameter maps^42^. Cardiac relaxometry is challenging as the relaxation times are calculated from a series of images that need to be acquired in the same cardiac and respiratory phase which can lead to long measurement times and can make the measurement sensitive to other types of patient motion. Here, T1 and T2 maps were measured with breath held bSSFP protocols, where the acquisition time was short enough to acquire all relevant information in a single breath hold, and additional motion compensation during post-processing allowed to correct for the cardiac motion. In some cases, T2 maps showed areas of lower T2 values in the posterior LV wall (as seen in Fig. 1D). This may be an artifact resulting from B_0_ inhomogeneities at the posterior wall that affect the T2 preparation pulses. Even though the artifact does not extend over the whole LCX region it may obscure potential T2 elevation due to the ischemia. However, T2 maps that were not affected by this artifact did not indicate any T2 elevation in this area.

T2* maps were measured using a breath held and ECG-triggered multi-echo FLASH sequence which is even less sensitive to residual motion. However, as the parameter T2* is sensitive to both microscopic field distortions caused by the iron-containing contrast agents and the meso- and macroscopic field variations as a consequence of magnetic susceptibility changes between tissues, this parameter is varying along the myocardium. To overcome this limitation, in this work T2* was measured before and after infusion of the molecular contrast agents; thus, the macroscopic field variations and a potential R2* increase due to the reperfusion injury (hemorrhage) were the same before and after administration, so that the measured R2* differences could solely be attributed to the contrast agent. However, small displacements can always lead to systematic errors in the R2* differences, which is a fundamental limitation of this technique.

As already discussed, we facilitated interventional MRI to selectively inject the contrast agents into the left coronary artery in order to reach sufficient concentration at the site of interest. Next to cost reductions, this approach added an additional internal control as it allowed to compare the lesion not only to remote healthy tissue (here: the area of the RCA), but also to the area of the LAD which was equally exposed to contrast agent, but not injured. Injection of medications via guiding catheters is already performed in clinical routine, and therefore injection of contrast agents is easily feasible from a technical point of view, allowing for high and immediate concentration of contrast agents in the vasculature.

A few limitations of the current study must be acknowledged. Despite all measures taken to optimize contrast deposition and imaging, the in vivo MRI signal still had a very low SNR with variations in absolute T2* times between single experiments. Therefore, comparison of absolute T2* times was impeded. Next to low binding efficiency of the compound under arterial shear stress, dislocation of the guiding catheter from the coronary ostium during injection of the contrast agent owing to anatomic variations of the pigs could have contributed to this finding. Forming an individual T2* relative ratio for the lesion and control area allowed us to compare results from individual experiments.

Microvascular structural changes in hypoperfused tissue are known to influence T2* times. Unspecific T2* signal changes in the lesion despite the absence of relevant moieties of MPIO as confirmed by histology most likely originated from such microscopic field distortions. Repetition of R2* = 1/T2* mapping ex vivo in a non-beating heart clearly depicted specific deposition of MPIO in the lesion as compared to the LAD and RCA area.

Last, injection of molecular contrast agents into the coronary artery does not fulfill the criteria of non-invasive imaging that would be favorable in this setting. In future approaches, increasing the signal of the contrast agent either by enhancing target specific binding or by using a contrast with an improved target to background ratio will be needed, e.g. Fluorine-19 which is selectively enhanced with a separate coil.

Our study represents a proof-of-concept study providing evidence that antibody-directed molecular imaging strategies using MRI are generally translatable from our previous experience in rodents to large animal models. By using real-time MRI-guided coronary interventions, target-specific imaging of early markers of inflammation was possible, providing a deeper understanding of post-ischemic cardiac lesions.

## Supplementary information

PigMI manuscript_301024-supplementary-information

Suplement Movie 2 post IR

Supplement Movie 1 - pre IR

## Data Availability

The datasets used and/or analyzed during the current study are available from the corresponding author on reasonable request.

## References

[1] Benjamin, E. J. et al. Heart Disease and Stroke Statistics-2019 Update: a report from the American Heart Association. *Circulation* **139**, e56–e528 (2019). 30700139 10.1161/CIR.0000000000000659

[2] Berezin, A. E. & Berezin, A. A. Adverse cardiac remodelling after acute myocardial infarction: old and new biomarkers. *Dis. Markers* **2020**, 1215802 (2020). 32626540 10.1155/2020/1215802 PMC7306098

[3] French, B. A. & Kramer, C. M. Mechanisms of post-infarct left ventricular remodeling. *Drug Discov. Today Dis. Mech.* **4**, 185–196 (2007). 18690295 10.1016/j.ddmec.2007.12.006 PMC2504336

[4] Matzinger, P. Tolerance, danger, and the extended family. *Annu. Rev. Immunol.* **12**, 991–1045 (1994). 8011301 10.1146/annurev.iy.12.040194.005015

[5] Blann, A. D., Nadar, S. K. & Lip, G. Y. The adhesion molecule P-selectin and cardiovascular disease. *Eur. Heart J.* **24**, 2166–2179 (2003). 14659768 10.1016/j.ehj.2003.08.021

[6] Panizzi, P. et al. Impaired infarct healing in atherosclerotic mice with Ly-6C(hi) monocytosis. *J. Am. Coll. Cardiol.* **55**, 1629–1638 (2010). 20378083 10.1016/j.jacc.2009.08.089 PMC2852892

[7] Nahrendorf, M., Pittet, M. J. & Swirski, F. K. Monocytes: protagonists of infarct inflammation and repair after myocardial infarction. *Circulation* **121**, 2437–2445 (2010). 20530020 10.1161/CIRCULATIONAHA.109.916346 PMC2892474

[8] Bohnen, S. et al. Tissue characterization by T1 and T2 mapping cardiovascular magnetic resonance imaging to monitor myocardial inflammation in healing myocarditis. *Eur. Heart J. Cardiovasc. Imaging* **18**, 744–751 (2017). 28329275 10.1093/ehjci/jex007

[9] Saeed, M., Liu, H., Liang, C. H. & Wilson, M. W. Magnetic resonance imaging for characterizing myocardial diseases. *Int. J. Cardiovasc. Imaging* **33**, 1395–1414 (2017). 28364177 10.1007/s10554-017-1127-x

[10] Nimura, A. et al. Site of transmural late gadolinium enhancement on the cardiac MRI coincides with the ECG leads exhibiting terminal QRS distortion in patients with ST-elevation myocardial infarctions. *Int. Heart J.* **53**, 270–275 (2012). 23038086 10.1536/ihj.53.270

[11] Jaffer, F. A., Sosnovik, D. E., Nahrendorf, M. & Weissleder, R. Molecular imaging of myocardial infarction. *J. Mol. Cell Cardiol.* **41**, 921–933 (2006). 17067633 10.1016/j.yjmcc.2006.09.008

[12] Jaffer, F. A., Libby, P. & Weissleder, R. Molecular imaging of cardiovascular disease. *Circulation* **116**, 1052–1061 (2007). 17724271 10.1161/CIRCULATIONAHA.106.647164

[13] Phinikaridou, A., Andia, M. E., Shah, A. M. & Botnar, R. M. Advances in molecular imaging of atherosclerosis and myocardial infarction: shedding new light on in vivo cardiovascular biology. *Am. J. Physiol. Heart Circ. Physiol.* **303**, H1397–H1410 (2012). 23064836 10.1152/ajpheart.00583.2012 PMC3532533

[14] Leuschner, F. & Nahrendorf, M. Molecular imaging of coronary atherosclerosis and myocardial infarction: considerations for the bench and perspectives for the clinic. *Circ. Res.* **108**, 593–606 (2011). 21372291 10.1161/CIRCRESAHA.110.232678 PMC3397211

[15] Lavin Plaza, B. et al. Molecular Imaging in Ischemic Heart Disease. *Curr. Cardiovasc. Imaging Rep.* **12**, 31 (2019). 31281564 10.1007/s12410-019-9500-x PMC6557873

[16] von zur Muhlen, C. et al. Magnetic resonance imaging contrast agent targeted toward activated platelets allows in vivo detection of thrombosis and monitoring of thrombolysis. *Circulation* **118**, 258–267 (2008). 18574047 10.1161/CIRCULATIONAHA.107.753657

[17] von Zur Muhlen, C. et al. A contrast agent recognizing activated platelets reveals murine cerebral malaria pathology undetectable by conventional MRI. *J. Clin. Invest.* **118**, 1198–1207 (2008). 18274670 10.1172/JCI33314 PMC2242620

[18] von Elverfeldt, D. et al. Dual-contrast molecular imaging allows noninvasive characterization of myocardial ischemia/reperfusion injury after coronary vessel occlusion in mice by magnetic resonance imaging. *Circulation* **130**, 676–687 (2014). 24951772 10.1161/CIRCULATIONAHA.113.008157

[19] Ridker, P. M., Cushman, M., Stampfer, M. J., Tracy, R. P. & Hennekens, C. H. Inflammation, aspirin, and the risk of cardiovascular disease in apparently healthy men. *N. Engl. J. Med.* **336**, 973–979 (1997). 9077376 10.1056/NEJM199704033361401

[20] MacRitchie, N., Noonan, J., Guzik, T. J. & Maffia, P. Molecular imaging of cardiovascular inflammation. *Br. J. Pharm.* **178**, 4216–4245 (2021). 10.1111/bph.15654 34378206

[21] Weil, B. R. et al. Brief myocardial ischemia produces cardiac Troponin I release and focal myocyte apoptosis in the absence of pathological infarction in swine. *JACC Basic Transl. Sci.* **2**, 105–114 (2017). 28979949 10.1016/j.jacbts.2017.01.006 PMC5624553

[22] Rumsey, W. L., Schlosser, C., Nuutinen, E. M., Robiolio, M. & Wilson, D. F. Cellular energetics and the oxygen dependence of respiration in cardiac myocytes isolated from adult rat. *J. Biol. Chem.* **265**, 15392–15402 (1990). 2394731

[23] Hausenloy, D. J. & Yellon, D. M. Myocardial ischemia-reperfusion injury: a neglected therapeutic target. *J. Clin. Invest.* **123**, 92–100 (2013). 23281415 10.1172/JCI62874 PMC3533275

[24] Botker, H. E., Kaltoft, A. K., Pedersen, S. F. & Kim, W. Y. Measuring myocardial salvage. *Cardiovasc. Res.* **94**, 266–275 (2012). 22311720 10.1093/cvr/cvs081

[25] Rurik, J. G., Aghajanian, H. & Epstein, J. A. Immune cells and immunotherapy for cardiac injury and repair. *Circ. Res.* **128**, 1766–1779 (2021). 34043424 10.1161/CIRCRESAHA.121.318005 PMC8171813

[26] Gentek, R. & Hoeffel, G. The innate immune response in myocardial infarction, repair, and regeneration. *Adv. Exp. Med. Biol.* **1003**, 251–272 (2017). 28667562 10.1007/978-3-319-57613-8_12

[27] Majmudar, M. D. et al. Monocyte-directed RNAi targeting CCR2 improves infarct healing in atherosclerosis-prone mice. *Circulation* **127**, 2038–2046 (2013). 23616627 10.1161/CIRCULATIONAHA.112.000116 PMC3661714

[28] Prabhu, S. D. & Frangogiannis, N. G. The biological basis for cardiac repair after myocardial infarction: from inflammation to fibrosis. *Circ. Res.* **119**, 91–112 (2016). 27340270 10.1161/CIRCRESAHA.116.303577 PMC4922528

[29] Moon, J. C. et al. Society for Cardiovascular Magnetic Resonance I, Cardiovascular Magnetic Resonance Working Group of the European Society of C. Myocardial T1 mapping and extracellular volume quantification: a Society for Cardiovascular Magnetic Resonance (SCMR) and CMR Working Group of the European Society of Cardiology consensus statement. *J. Cardiovasc Magn. Reson* **15**, 92 (2013). 24124732 10.1186/1532-429X-15-92 PMC3854458

[30] O’Brien, A. T., Gil, K. E., Varghese, J., Simonetti, O. P. & Zareba, K. M. T2 mapping in myocardial disease: a comprehensive review. *J. Cardiovasc. Magn. Reson.* **24**, 33 (2022). 35659266 10.1186/s12968-022-00866-0 PMC9167641

[31] Ferreira, V. M., Piechnik, S. K., Robson, M. D., Neubauer, S. & Karamitsos, T. D. Myocardial tissue characterization by magnetic resonance imaging: novel applications of T1 and T2 mapping. *J. Thorac. Imaging* **29**, 147–154 (2014). 24576837 10.1097/RTI.0000000000000077 PMC4252135

[32] Jia, Z. et al. Detection of acute myocarditis using T1 and T2 mapping cardiovascular magnetic resonance: a systematic review and meta-analysis. *J. Appl. Clin. Med. Phys.* **22**, 239–248 (2021). 34480832 10.1002/acm2.13365 PMC8504590

[33] Arai, A. E. Magnetic resonance imaging for area at risk, myocardial infarction, and myocardial salvage. *J. Cardiovasc. Pharm. Ther.* **16**, 313–320 (2011). 10.1177/1074248411412378 PMC8690274 21821534

[34] Gotsch, U., Jager, U., Dominis, M. & Vestweber, D. Expression of P-selectin on endothelial cells is upregulated by LPS and TNF-alpha in vivo. *Cell Adhes. Commun.* **2**, 7–14 (1994). 7526954 10.3109/15419069409014198

[35] McEver, R. P. Selectins: initiators of leucocyte adhesion and signalling at the vascular wall. *Cardiovasc. Res.* **107**, 331–339 (2015). 25994174 10.1093/cvr/cvv154 PMC4592324

[36] Chen, M. & Geng, J. G. P-selectin mediates adhesion of leukocytes, platelets, and cancer cells in inflammation, thrombosis, and cancer growth and metastasis. *Arch. Immunol. Ther. Exp. (Warsz.)* **54**, 75–84 (2006). 16648968 10.1007/s00005-006-0010-6

[37] Sommer, P. et al. Increasing soluble P-selectin levels predict higher peripheral atherosclerotic plaque progression. *J. Clin. Med.* **12** (2023). 10.3390/jcm12206430 PMC10607706 37892568

[38] von Zur Muhlen, C. et al. Functionalized magnetic resonance contrast agent selectively binds to glycoprotein IIb/IIIa on activated human platelets under flow conditions and is detectable at clinically relevant field strengths. *Mol. Imaging* **7**, 59–67 (2008). 18706288 PMC2912508

[39] Maier, A. et al. Molecular magnetic resonance imaging of activated platelets allows noninvasive detection of early myocarditis in mice. *Sci. Rep.* **10**, 13211 (2020). 32764735 10.1038/s41598-020-70043-9 PMC7413393

[40] Bauer, W. et al. Theory of coherent and incoherent nuclear spin-dephasing in the heart. *Phys. Rev. Lett.* **83** (1999).

[41] Wacker, C. M. et al. BOLD-MRI in ten patients with coronary artery disease: evidence for imaging of capillary recruitment in myocardium supplied by the stenotic artery. *MAGMA* **8**, 48–54 (1999). 10383093 10.1007/BF02590635

[42] Hosch, W. et al. MR-relaxometry of myocardial tissue: significant elevation of T1 and T2 relaxation times in cardiac amyloidosis. *Invest. Radio.* **42**, 636–642 (2007). 10.1097/RLI.0b013e318059e021 17700279

