# Supplementary material for: Interventional real-time molecular MRI for targeting early myocardial injury in a pig model
Source: Npj Imaging. 2025 Feb 3;3:7. doi: 10.1038/s44303-025-00069-z (PMC12118736; doi:10.1038/s44303-025-00069-z)
Supplement: Supplementary file 1 — PigMI manuscript_301024-supplementary-information [file 44303_2025_69_MOESM1_ESM.pdf]

Interventional real-time molecular MRI for targeting early myocardial injury in a pig model

Supplement material

Supplement figure 1: Timeline

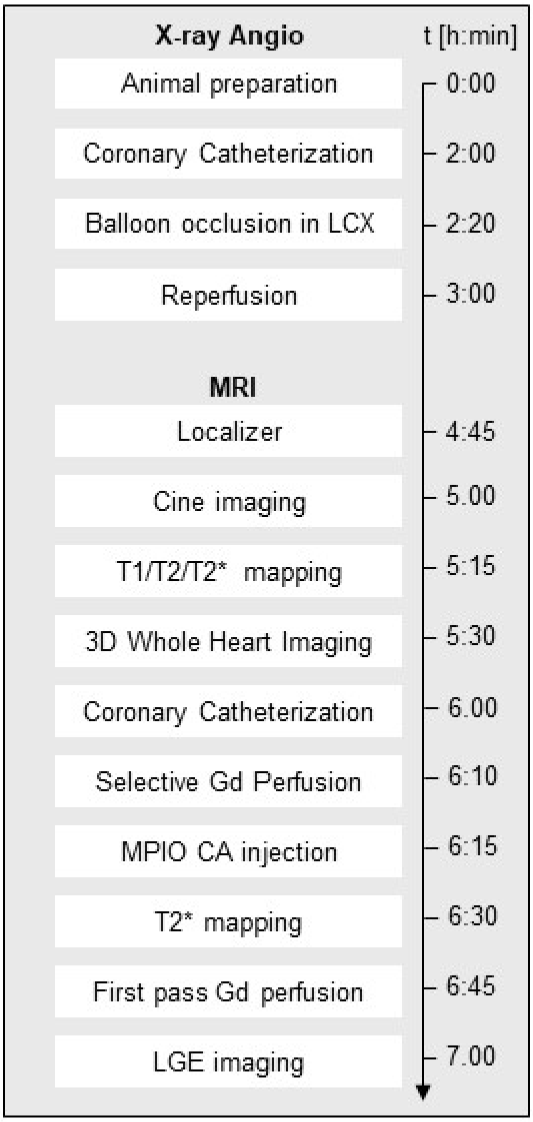

Supplement figure 1 *Timeline of the animal experiment starting with the creation of a ischemic lesion under X-ray imaging. Subsequently, the animal is transferred to the MRI, where initial localizer images and relaxometry measurements are performed. Once the catheter is introduced into the target coronary artery, selective perfusion measurements are acquired using conventional Gd-based CAs followed by the injection of the MPIO CA. After the injection relaxometry is performed together with an LGE acquisition.*

Legend supplement movies:

Supplement Movie 1 pre IR.mov
Echocardiography of the left ventricle in a short axis view before ischemia/reperfusion

Supplement Movie 2 post IR.mov
Echocardiography of the left ventricle in a short axis view after ischemia/reperfusion
